# Recurrent Sacral Chordoma: A Case Report

**DOI:** 10.31729/jnma.5401

**Published:** 2020-11-30

**Authors:** Abhash Shrestha, Prami Nakarmi, Animesh Nanda Vaidya, Sumit Raut, Binod Rajbhandari, Mohan Raj Sharma

**Affiliations:** 1 Chirayu National Hospital and Medical Institute, Basundhara, Kathmandu, Nepal; 2 Venus Hospital, Mid Baneshwor, Kathmandu, Nepal; 3 Kathmandu Medical College, Sinamangal, Kathmandu, Nepal

**Keywords:** *chordoma*, *rare disease*, *recurrent*, *sacral chordoma*, *sacrum*

## Abstract

Chordoma is a rare and locally aggressive tumor that arises from the notochordal remnants and has an incidence of 0.1/100000 per year. It has a predilection for the axial skeleton and is the most common primary malignant tumor of sacrum. The mainstay of treatment is wide surgical excision but there is a risk of recurrence due to the infiltrating nature of the tumor. Here, we report a case of a 56-years male who complained of pain over his sacral region for the past two years with episodic urinary symptoms, constipation, and weakness of both legs. Seven years after undergoing surgery and radiotherapy for his sacral chordoma, he was diagnosed with recurrent sacral chordoma and planned for reoperation. Subtotal excision of the chordoma was done which significantly alleviated his symptoms postoperatively. Timely intervention helps to improve the quality of life in patients with either primary or recurrent sacral chordomas.

## INTRODUCTION

Chordoma is a rare, slow-growing malignant bone tumor arising from remnants of the notochord.^1^ Sacral chordomas represent half of all chordomas and are commonly diagnosed in the fifth to sixth decades of life.^1,2^ The main treatment is wide surgical excision which is often associatedwith complications related to sacral nerve root injury.^1^ Inadequate surgical resection due to anatomical limitations and wound infiltration by seeding tumor cells from pseudocapsule results in recurrence generally after four years.^1,3,4^ We present a case of a 56-year male patient diagnosed with recurrent sacral chordoma who presented with symptoms, five years after surgical and radiotherapeutic intervention.

## CASE REPORT

A 56-years man with a two-year history of pain and numbness over his sacral region was admitted under the neurosurgery department in our hospital. He had a sharp and shooting type of pain that radiated from his lower back to his right foot. The severity of pain gradually increased that limited his day-to-day activities. He also complained of constipation, increased frequency of micturition, and urge incontinence throughout the last two years.Over the last six months, he also developed weakness of both of his lower limbs and was neither able to walk without support nor stand for more than fifteen minutes. The rest of the history was only significant for diabetes mellitus under control by oral hypoglycemic drugs.

He had undergone subtotal resection of the sacral chordoma seven years back for similar symptoms in another institution but by the same neurosurgeon (MRS). The patient had subsequently received postoperative radiotherapy and was asymptomaticuntil the present complaints about two years ago.

**Figure 1. (A, B).**
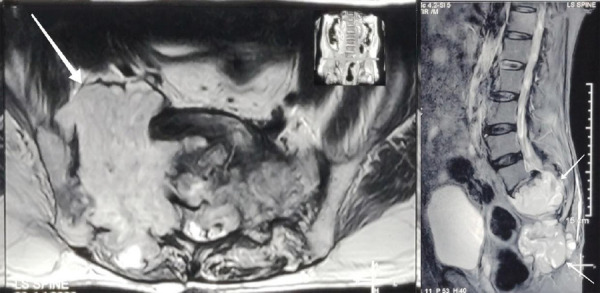
Axial section of MRI of the LS spine with a huge expansile mass in the upper sacrum (white arrow) (A), Sagittal section of T2 weighted MRI of the LS spine showing the mass (white arrows) (B).

On neurological examination, he had reduced power in the lower limbs about 3/5 on the right side and 4/5 on the left side; however, sensation and reflexes were normal. The straight leg raise test was 60 degrees on the right leg and 20 degrees on the left leg. Rectal examination revealed normal anal tone and deep anal pressure sensation. The rest of the physical and systemic examinations were within normal limits. A magnetic resonance imaging (MRI) of the lumbosacral (LS) spine (Figure 1) was obtained which revealed a large mass in both ala of the sacrum; which when compared to a year-old scan, corroborated that the overall size of the tumor had increased. The mass on the right side of the sacrum had an exophytic component measuring approximately 11.5 cm craniocaudally (CC), 7.7 cm transversely (T), and 4.5 cm anteroposteriorly (AP) while the mass extending to the left sacral wing measured 4.6cm (CC) X 8.1cm (T) X 4.9cm (AP). A diagnosis of recurrent sacral chordoma with diabetes mellitus was made.

**Figure 2:**
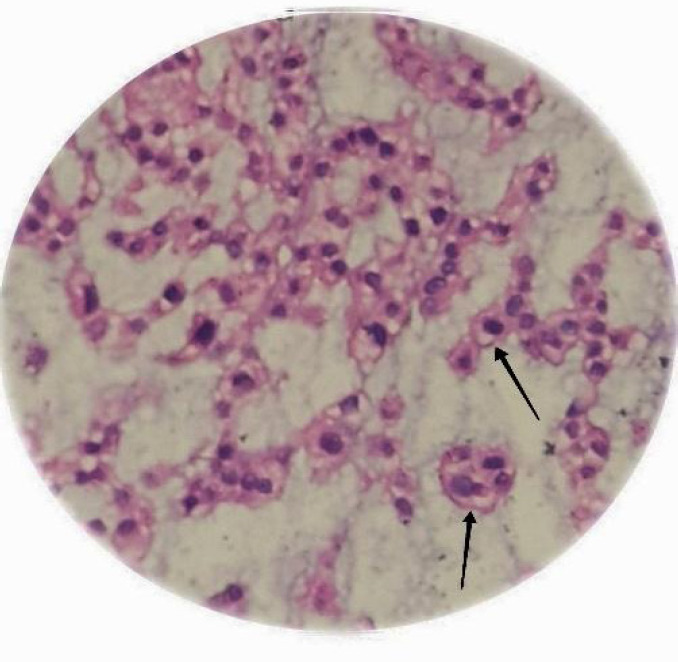
Photomicrograph of the histopathological slide showing the tumor cells having an indistinct cell border with abundant eosinophilic to vacuolated cytoplasm (black arrows) and centrally placed nuclei (HE Stain, X100).

The option of re-operation was offered to the patient given his progressive symptoms. Under general anesthesia in the prone position, an inverted-V incision was made over the previous scar mark. The tumor was exposed superiorly and inferiorly and all the visible mass was excised and the wound was closed in layers. He tolerated the surgery well without any untoward event in the postoperative period. He was discharged on the eighth postoperative day and his sutures were removed on the fourteenth postoperative day. The histopathological report confirmed the diagnosis of chordoma (Figure 2).

The patient's pain had significantly diminished postoperatively and his power had increased to 5/5 in both of his legs at two months follow-up. He is due to receive radiotherapy shortly.

## DISCUSSION

Sacral chordomas usually reach considerable size before they are diagnosed as the tumors often grow significantly before damaging critical structures.^5^ Almost all patients with sacral chordoma present with persistent low back pain; usually in the 5th-6th decades of their lives.^1^ Some patients may have an added radicular component to their pain due to compression of the sciatic nerve or iliolumbar trunk, which may be steadily treated as non-specific sciatica.^5^ One-third of patients may develop urinary tract infections and up to 10% of patients will have constipation or features of cauda equina symptoms.^1^ The median time from initial symptom to diagnosis is nearly two years due to these vague symptoms.^6^ In our case, the patient was diagnosed with sacral chordoma four years after his initial symptom.

Surgery is the mainstay of treatment while other options include radiotherapy and medical therapy.^2^ Surgery can be done via the anterior and/or posterior approach; however, an adequate surgical margin can be achieved in only about 50% of cases of sacral chordoma due to the anatomical constraints and technical limitations.^1,2,7^ Inadequate surgical margins or contamination of the surgical wound via tumor cell seeding from the ruptured pseudo-capsule will inevitably result in poorly salvageable locoregional recurrences.^2,3^ The posterior only approach has shown to be more favorable considering the significant patient morbidity and mortality caused by the combined approach without any significant improvement in recurrence rate and long term survival.^1^ En-bloc resection with sacrectomy provides good long-term tumor control but at the expense of substantial perioperative morbidity including impairment of bowel, bladder, and motor functions.^2^ Radiotherapy should be considered as an adjuvant treatment after surgical excision as it provides an overall positive effect on overall survival (OS), local relapse-free survival (LRFS), and distant metastasis-free survival (DMFS) in patients presenting with either a primary sacral chordoma or a first locally recurrent sacral chordoma.^4^ Cytotoxic drugs are not recommended, however, tyrosine kinase inhibitors (TKI) may help to achieve a stable disease state in patients with widespread or recurrent chordoma who cannot tolerate further surgical or radiotherapeutic intervention.^8^

The recurrence of chordoma in patients who underwent subtotal excision is up to three times higher when compared to patients who underwent radical excision.^3^ In a retrospective study by Yang Y et al, sacral chordoma recurred in 31.3%, 24.2%, and 6.5% of patients who underwent intracapsular resection, marginal resection, and wide resection respectively whereas the median recurrence-free survival time was 47 months, 65 months and 124 months respectively.^6^ In the same study, the median LRFS time was 69±12.7 months after any kind of surgical intervention.^6^ In another retrospective study where the patients received radiotherapy after surgery, the LRFS at five years was 86% and the median LRFS time was 51 months.^4^ Our patient had a recurrence-free time of five years.

For recurring sacral chordomas, the treatment options are the same as that of primary sacral chordomasand if the patient is compliant, surgical resection with adjuvant radiotherapy should be preferred.^4^ In the study by van Wulfften Palthe OD et al, patients who underwent surgery for their first recurrent sacral chordomas, the OS, LRFS, and DMFS at five years was 65%, 79%, and 64% respectively whereas the median OS time, LRFS time, and DMFS time was 86 months, 44 months, and 30 months respectively.^4^ Metastasis may occur in the lungs, liver, bone, skin, regional lymph nodes, and other sites and is seen in about 30-40% of patients in the late stages of their disease if there is evidence of local recurrence.^2^ In the study by Yang Y et al., metastasis was seen in 21.6% of patients who had local recurrence.^6^

The insidious course of chordoma unwantedly results in late diagnosis, by when swift intervention is often needed. Sacral chordomas and their recurrences, though rare, can be managed surgically mainly for the symptomatic relief and for prolonging survival. Strict clinical follow-up and serial imaging to monitor any increase in the size of the tumor, and timely intervention are of paramount importance in patients with recurrent sacral chordomas so that they may have a good balance between morbidity and quality of life.
